# Charts and LMS Tables of Transfontanellar and Transvertical Ear-to-Ear Distances for Gestational Age

**DOI:** 10.3389/fped.2022.838333

**Published:** 2022-05-04

**Authors:** Nancy Arnold, Rudolf Georg Ascherl, Ulrich Herbert Thome

**Affiliations:** Division of Neonatology, Center for Pediatric Research, University of Leipzig, Leipzig, Germany

**Keywords:** anthropometry, infant, premature, head growth, cephalometry, ear-to-ear distance, centile charts, LMS tables

## Abstract

**Introduction:**

To date cranial development has only been described by analyzing occipitofrontal circumference (OFC). More precise methods of determining head measurements have not been widely adopted. The use of additional measurements has the potential to better account for the three-dimensional structure of the head. Our aim was to put forward centile curves of such measurements for gestational age along with a compound head volume index.

**Methods:**

We created generalized additive models for location, scale, and shape of two ear-to-ear distances (EED), transfontanellar (fEED) and transvertical (vEED), from birth anthropometric data. Same was done for OFC, crown-heel length, and birth weight to allow for comparison of our models with growth charts by Voigt et al. and Fenton and Kim.

**Results:**

Growth charts and tables of LMS parameters for fEED and vEED were derived from 6,610 patients admitted to our NICU and 625 healthy term newborns. With increasing gestational age EEDs increase about half as fast compared to OFC in absolute terms, their relative growths are fairly similar.

**Discussion:**

Differences to the charts by Fenton and Kim are minute. Tape measurements, such as fEED or vEED can be added to routine anthropometry at little extra costs. These charts may be helpful for following and evaluating head sizes and growth of preterm and term infants in three dimensions.

## Introduction

One of the main aims of preterm infant care is to mitigate the impact of preterm birth on the crucial steps of brain development otherwise taking place *in utero*. Tracking head growth, particularly from birth to term, has been associated with attaining this goal (1). While there are more precise methods of determining head volume (2–6), the tape measure is superior to all of them in effortlessness, availability and safety. However, occipitofrontal head circumference (OFC) is a two-dimensional measure, disregarding vertical head growth. Some of the discrepancies between OFC and brain volume have been shown to be reduced by taking additional measurements into account (5). Ear-to-ear distances (EED) can be measured as easily as OFC and account for the third dimension of head growth. However, the lack of reference values limits the clinical use of additional head measurements. Herein, we set out to close this gap and calculate percentile curves of EED measurements.

## Patients and Methods

### Aim

In this study, we have analyzed two different methods for EED measurements taken at birth in relation to gestational age to calculate normative charts.

### Patients and Setting

Patients were recruited at the University of Leipzig Medical Center, a large tertiary care perinatal center in the German state of Saxony. It has the highest number of deliveries of all hospitals in the state (7) and a large proportion of high-risk deliveries. Each year an average of 108 preterms with birth weights below 1,500 g were admitted over the last 5 years (8). Anthropometric measurements came from two sources: (a) We measured healthy term babies at our maternity ward after informed consent between June 2017 and May 2019 and (b) extracted the first anthropometric data obtained within the first 72 h of life of all admissions to our neonatal intensive care unit (NICU) from routine electronic patient records from 2007 to 2020. Birth date and gestational age were taken from patient records. We excluded patients with inborn deformities of the scull and congenital intracranial hemorrhage. The study protocol was approved of by the institutional review board of the Medical Faculty at the University of Leipzig (internal reference number 168/17-ek).

### Measurements

**Figure 1:**
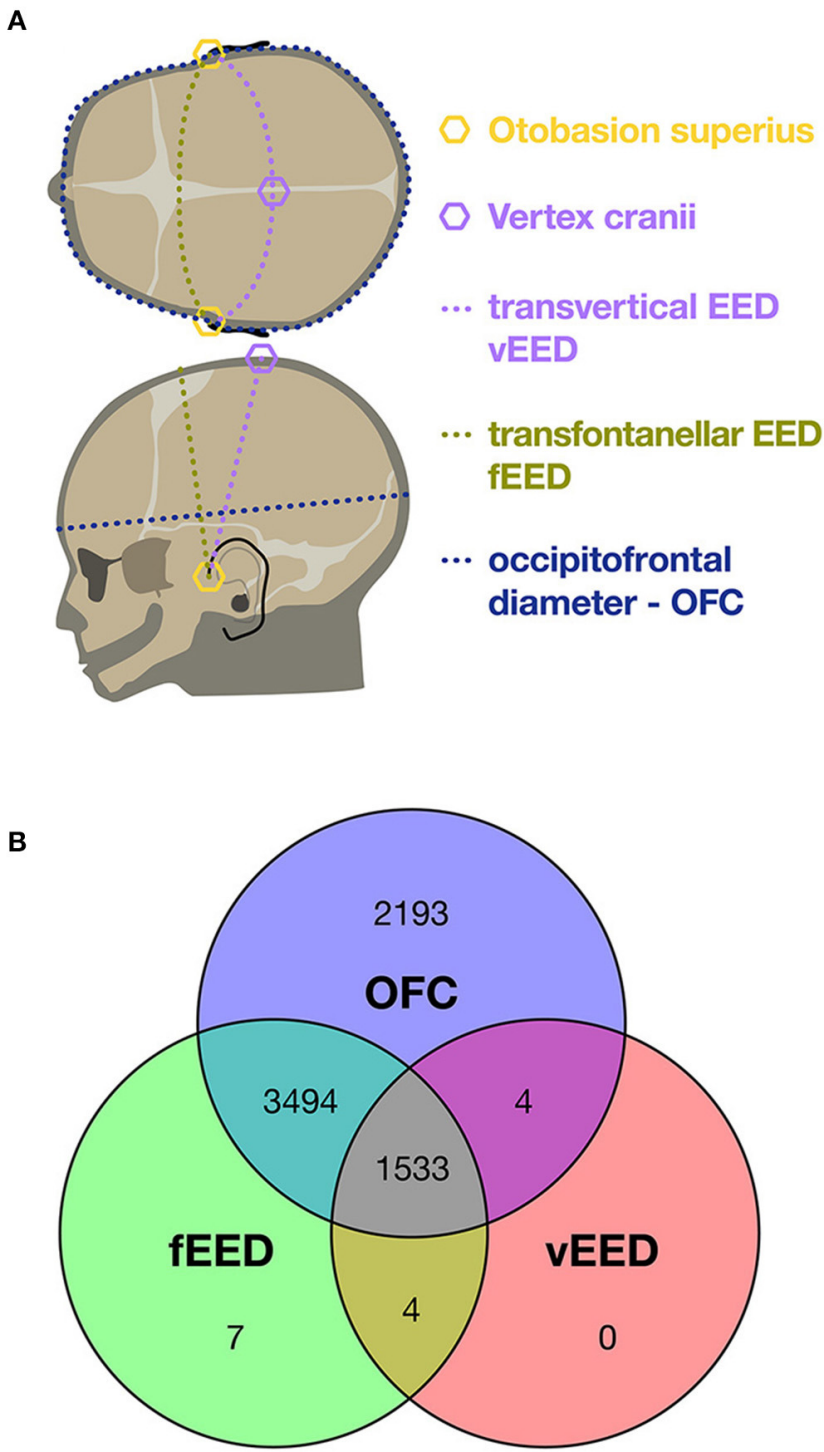
**(A)** Schematic of cranial measures. **(B)** Venn diagram of the 13,803 head circumferences acquired from the 7,235 patients included. In 69% occipitofrontal circumference (OFC) and transfontanellar ear-to-ear distance (EED) were available, in 21% all three head circumferences. This is mostly due to the fact that transvertical ear-to-ear distances (vEED) was added to the protocol during the course of the study.

In addition to the crown-heel length (CHL), 3 head measurements were obtained with a flexible tape measure to an accuracy of 5 mm. These were OFC and two distinct EEDs (see Figure 1A): The transfontanellar EED (fEED) was defined as the distance from the superior insertion of one ear (otobasion superius, OBS) over the large fontanella to the other OBS. The transvertical EED (vEED) was measured over the vertex of the head also starting from and ending at the OBS. Birth weights were determined routinely by midwifes and nursing staff with calibrated scales and were rounded to the nearest 5 g. All measurements were taken within 72 h after birth.

### Head Volume Index

Likening the head to an ellipsoid, its volume would be proportional to its three principal diameters: OFC measured around the base of the neurocranium would represent two of these and an EED the third. We thus propose a head volume index HVI = OFC^2^ × vEED that we subjected to the same analyses as the EEDs.

### Data Analysis, Statistics and Visualization

We fit generalized additive models for location, scale, and shape (GAMLSS) to our data using the method by Cole and Green (9) which returns parameters for location (median, μ), scale (variance, σ) and shape (Box-Cox power, λ). These Greek letters gave rise to the denomination Lambda Mu Sigma (LMS) method. The curves were smoothed by means of penalized beta splines. This method has been utilized and recommended by the World Health Organization (WHO) (10, 11), Robert Koch-Institut, Berlin, Germany (RKI) (12), and Fenton and Kim (13). For this purpose and all other analyses we used the R software environment (10) in conjunction with the GAMLSS package (14, 15). To compare our results to those of Fenton and Kim (13) we traced their growth curves by using WebPlotDigitizer (16), because percentiles and LMS data were not available.

## Results

### Patient Characteristics

We included a total 7,235 neonates of which 625 were healthy term babies on our maternity ward and 6,610 patients from our NICU. Forty two percent were female. The NICU set contained few implausible data: We excluded 3 OFC values, 42 fEED, and 216 vEED. These had a median absolute value of z-scores of 5.66 (IQR 5.05–6.27) compared against the models described below; the z-score distributions of the included and excluded values are charted in Supplementary Figure 1. A total of 5,038 fEED and 1,541 vEED measurements were included, see Figure 1B.

### Charts and LMS Tables

**Figure 2:**
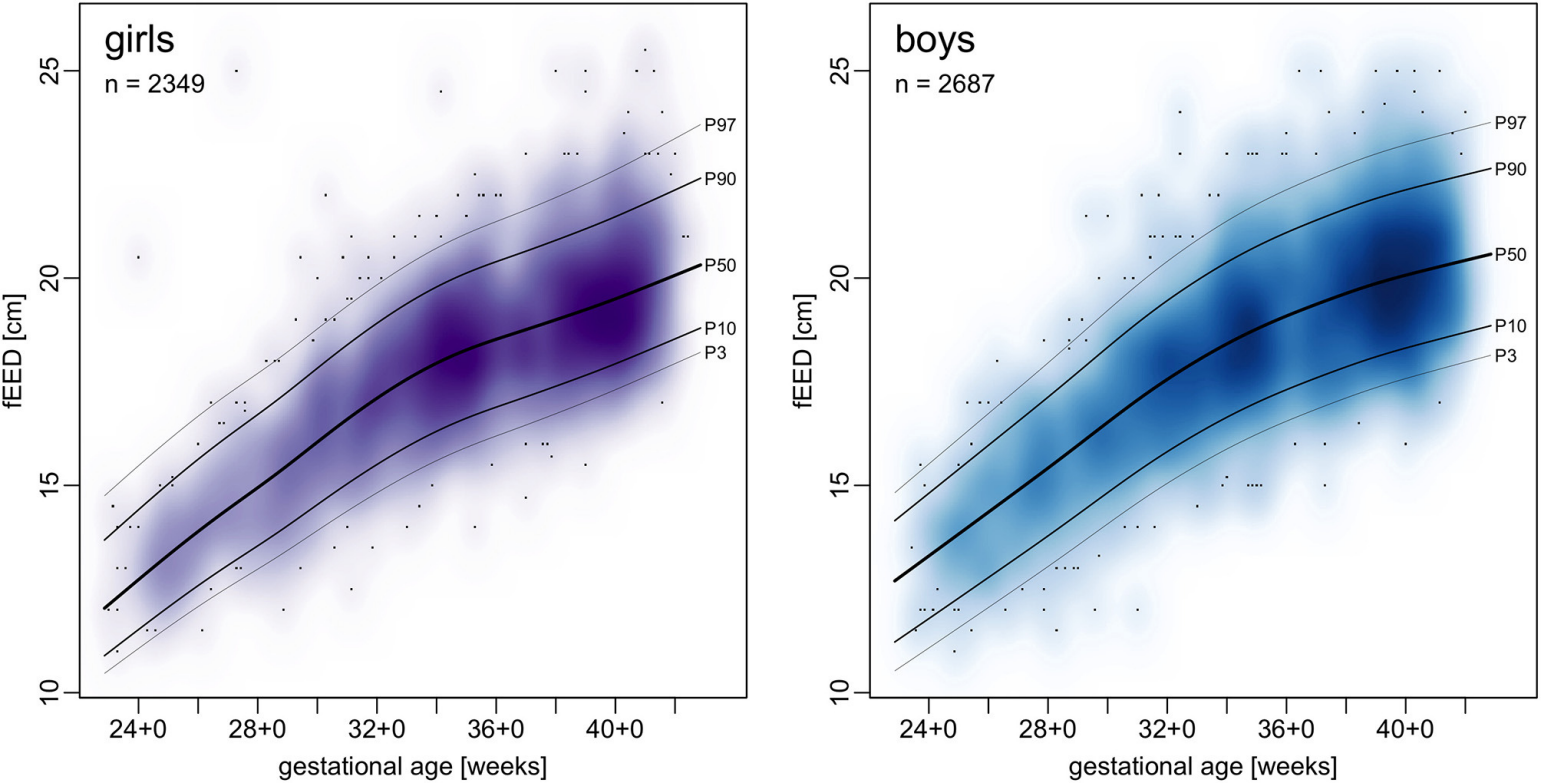
Charts of transfontanellar ear-to-ear distance (fEED) in relation to gestational age.

**Figure 3:**
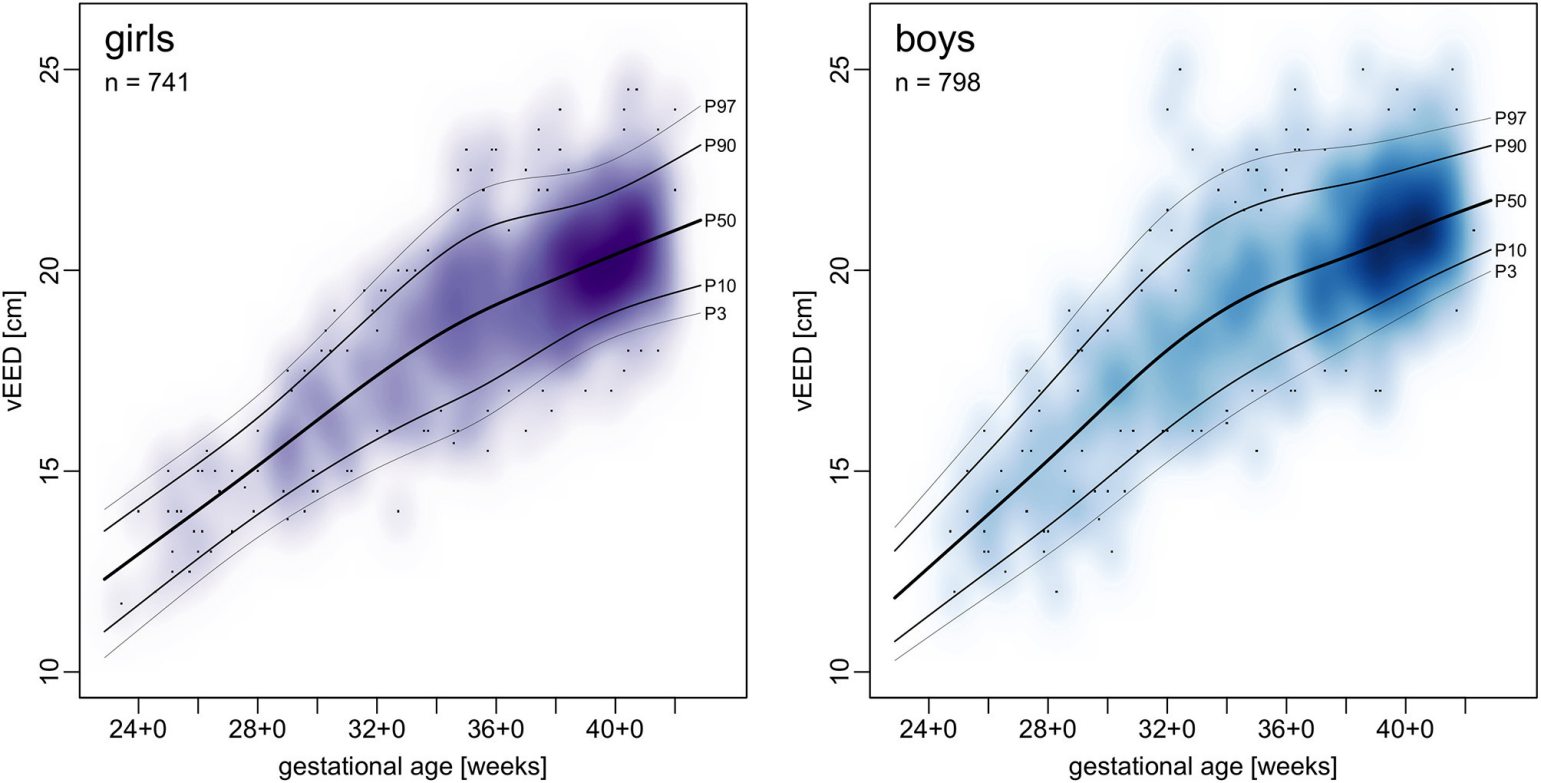
Charts of transvertical ear-to-ear distance (vEED) in relation to gestational age.

Centile curves were plotted over kernel density estimations of EED (Figures 2, 3). Graded charts are supplemented to this article (see Supplementary Figure 2). Tables 1–4 show model parameters and centile estimations for completed weeks of gestation, tables for OFC are supplemented (Supplementary Tables 1, 2).

**Table 1:** LMS parameters of models of transfontanellar EED (fEED) for girls.

**GA**	**P3**	**P10**	**P50 = M**	**P90**	**P97**	**S**	**L**	**n**
23	10.54	10.97	12.12	13.77	14.84	0.0870	−2.1754	9
24	11.07	11.52	12.73	14.41	15.47	0.0859	−2.0225	33
25	11.58	12.06	13.32	15.04	16.09	0.0848	−1.8560	44
26	12.08	12.58	13.89	15.63	16.67	0.0837	−1.6657	38
27	12.54	13.07	14.43	16.18	17.20	0.0826	−1.4424	45
28	12.98	13.54	14.94	16.71	17.70	0.0816	−1.1825	64
29	13.43	14.03	15.48	17.25	18.22	0.0805	−0.9051	83
30	13.91	14.54	16.04	17.83	18.77	0.0795	−0.6414	98
31	14.38	15.04	16.59	18.39	19.32	0.0785	−0.4146	115
32	14.82	15.50	17.10	18.91	19.84	0.0775	−0.2483	122
33	15.23	15.93	17.56	19.38	20.31	0.0765	−0.1635	165
34	15.60	16.30	17.95	19.79	20.72	0.0755	−0.1674	221
35	15.91	16.62	18.27	20.12	21.07	0.0746	−0.2546	215
36	16.19	16.89	18.52	20.39	21.36	0.0736	−0.4186	113
37	16.46	17.14	18.76	20.65	21.64	0.0727	−0.6537	150
38	16.73	17.39	18.99	20.91	21.94	0.0717	−0.9460	219
39	17.01	17.65	19.24	21.19	22.26	0.0708	−1.2751	239
40	17.31	17.93	19.50	21.48	22.61	0.0699	−1.6224	263
41	17.62	18.23	19.78	21.80	22.98	0.0690	−1.9747	107
42	17.94	18.53	20.07	22.12	23.37	0.0682	−2.3250	6

**Table 2:** LMS parameters of models of transfontanellar EED (fEED) for boys.

**GA**	**P3**	**P10**	**P50 = M**	**P90**	**P97**	**S**	**L**	**n**
23	10.59	11.29	12.77	14.23	14.91	0.0897	1.1070	6
24	11.08	11.79	13.30	14.81	15.52	0.0887	1.0025	29
25	11.56	12.28	13.83	15.39	16.12	0.0877	0.8979	53
26	12.05	12.78	14.35	15.97	16.73	0.0867	0.7934	56
27	12.55	13.28	14.88	16.55	17.34	0.0857	0.6888	78
28	13.04	13.78	15.42	17.13	17.96	0.0847	0.5843	79
29	13.55	14.30	15.97	17.73	18.58	0.0837	0.4797	84
30	14.07	14.82	16.52	18.33	19.22	0.0828	0.3752	113
31	14.58	15.34	17.06	18.92	19.83	0.0818	0.2706	137
32	15.05	15.81	17.56	19.46	20.40	0.0809	0.1661	171
33	15.48	16.25	18.01	19.94	20.91	0.0800	0.0615	154
34	15.88	16.64	18.41	20.38	21.37	0.0790	−0.0430	238
35	16.23	17.00	18.77	20.76	21.78	0.0781	−0.1476	194
36	16.55	17.31	19.09	21.10	22.13	0.0772	−0.2521	126
37	16.84	17.59	19.37	21.40	22.44	0.0763	−0.3567	195
38	17.11	17.86	19.63	21.67	22.73	0.0755	−0.4612	224
39	17.36	18.10	19.87	21.92	22.99	0.0746	−0.5658	310
40	17.58	18.31	20.07	22.13	23.21	0.0737	−0.6703	287
41	17.78	18.50	20.25	22.31	23.41	0.0729	−0.7749	150
42	17.97	18.69	20.43	22.49	23.60	0.0720	−0.8794	3

**Table 3:** LMS parameters of models of transvertical EED (vEED) for girls.

**GA**	**P3**	**P10**	**P50 = M**	**P90**	**P97**	**S**	**L**	**n**
23	10.44	11.09	12.39	13.59	14.12	0.0784	1.7816	1
24	11.05	11.67	12.93	14.12	14.66	0.0738	1.6549	2
25	11.65	12.25	13.48	14.65	15.19	0.0695	1.5283	9
26	12.25	12.82	14.03	15.19	15.72	0.0657	1.4016	8
27	12.82	13.38	14.57	15.74	16.28	0.0631	1.2749	4
28	13.35	13.92	15.13	16.33	16.88	0.0621	1.1482	11
29	13.84	14.43	15.70	16.96	17.56	0.0630	1.0215	23
30	14.28	14.91	16.27	17.64	18.28	0.0654	0.8948	22
31	14.69	15.37	16.83	18.33	19.04	0.0687	0.7682	18
32	15.07	15.79	17.38	19.02	19.80	0.0725	0.6415	26
33	15.41	16.18	17.89	19.68	20.55	0.0763	0.5148	31
34	15.74	16.55	18.36	20.30	21.24	0.0796	0.3881	56
35	16.08	16.91	18.78	20.81	21.81	0.0810	0.2614	64
36	16.50	17.31	19.15	21.15	22.14	0.0781	0.1347	35
37	17.01	17.76	19.47	21.35	22.29	0.0718	0.0081	58
38	17.53	18.21	19.78	21.50	22.36	0.0648	−0.1186	94
39	17.99	18.63	20.09	21.69	22.50	0.0594	−0.2453	111
40	18.34	18.97	20.40	21.98	22.77	0.0575	−0.3720	113
41	18.59	19.23	20.70	22.34	23.18	0.0586	−0.4987	53
42	18.78	19.44	20.99	22.75	23.66	0.0612	−0.6254	2

**Table 4:** LMS parameters of models of transvertical EED (vEED) for boys.

**GA**	**P3**	**P10**	**P50 = M**	**P90**	**P97**	**S**	**L**	**n**
23	10.36	10.84	11.94	13.13	13.72	0.0746	0.1698	0
24	10.88	11.40	12.60	13.91	14.56	0.0774	0.1242	2
25	11.39	11.96	13.26	14.69	15.41	0.0803	0.0787	6
26	11.90	12.51	13.93	15.49	16.28	0.0833	0.0331	10
27	12.41	13.07	14.59	16.30	17.17	0.0863	−0.0125	14
28	12.93	13.63	15.27	17.12	18.07	0.0890	−0.0581	15
29	13.48	14.22	15.97	17.96	18.98	0.0911	−0.1036	20
30	14.05	14.84	16.68	18.79	19.88	0.0921	−0.1492	24
31	14.65	15.45	17.37	19.57	20.72	0.0922	−0.1948	23
32	15.23	16.05	18.01	20.27	21.45	0.0911	−0.2404	33
33	15.78	16.61	18.58	20.85	22.04	0.0888	−0.2860	37
34	16.30	17.12	19.06	21.31	22.48	0.0854	−0.3315	41
35	16.78	17.57	19.46	21.63	22.77	0.0810	−0.3771	34
36	17.22	17.98	19.78	21.85	22.93	0.0760	−0.4227	35
37	17.64	18.36	20.06	22.00	23.01	0.0705	−0.4683	68
38	18.06	18.74	20.33	22.14	23.08	0.0651	−0.5138	89
39	18.48	19.12	20.62	22.31	23.18	0.0602	−0.5594	146
40	18.90	19.51	20.93	22.51	23.33	0.0559	−0.6050	128
41	19.30	19.88	21.23	22.73	23.49	0.0522	−0.6506	71
42	19.67	20.23	21.51	22.93	23.65	0.0489	−0.6961	2

vEED was larger than fEED in 82% of the patients in which both measurements had been taken (difference 1.02 ± 1.21 cm, *t*_(3052.2)_ = 13.523, *p*-value < 2.2 <10^−16^).

EEDs are not just generally about 40 % smaller than OFC are but also grow only about half (0.50-fold to 0.66-fold) as fast as OFC per time interval in absolute terms. Their relative growth rates, however, closely resemble one another (see Supplementary Figure 3).

### Head Volume Index

**Figure 4:**
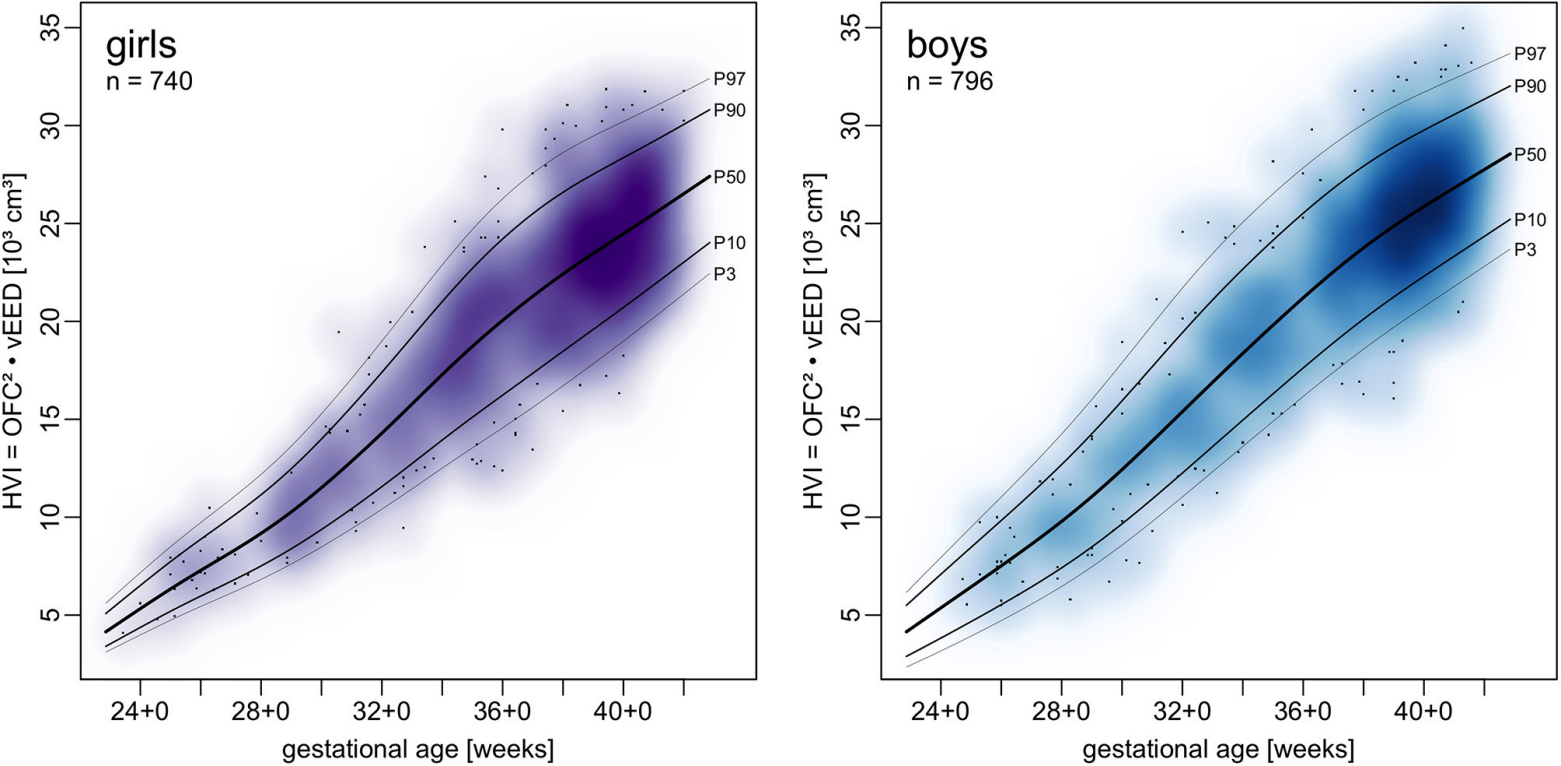
Charts of a head volume index (HVI) combining OFC and vEED in relation to gestational age.

Centile curves and LMS tables of HVI are given in Figure 4 and Supplementary Tables 3, 4.

## Discussion

To the best of our knowledge these are the first charts of intrauterine EED growth for gestational ages. Since preterm infant growth is frequently assessed by comparing it to intrauterine growth, these charts provide a new tool for assessing head growth in all three dimensions.

**Figure 5:**
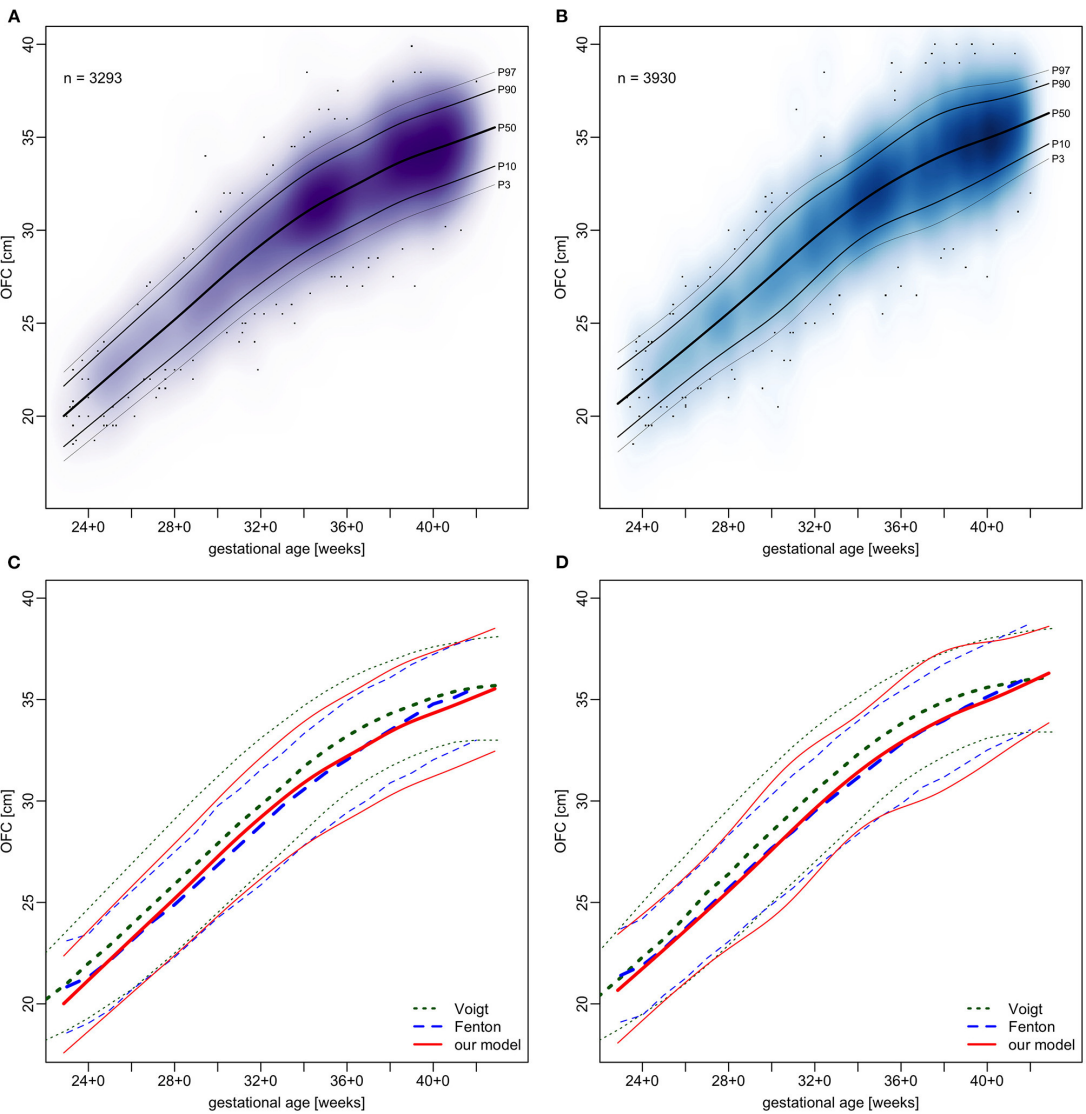
Charts of occipitofrontal circumference (OFC) in relation to gestational age. **(A,B)** our sample with fitted models. **(C**,**D)** comparison of our models (red) with Voigt et al. (17) (green) and Fenton and Kim (13) (blue), shown are 3rd, 50th, and 97th percentiles. **(A,C)** girls and **(B,D)** boys.

**Figure 6:**
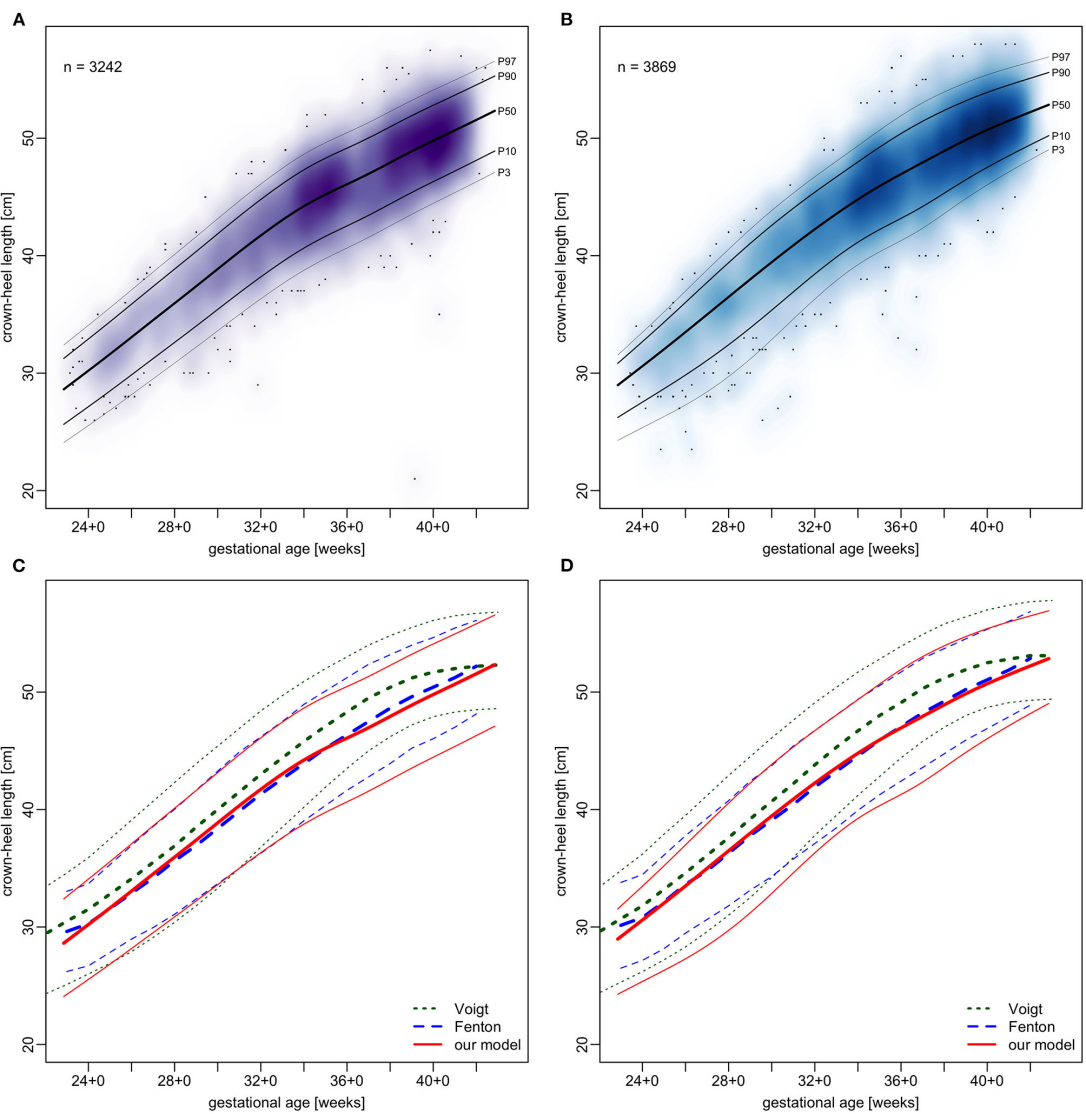
Charts of crown-heel length in relation to gestational age. **(A,B)** our sample with fitted models. **(C,D)** comparison of our models (red) with Voigt et al. (17) (green) and Fenton and Kim (13) (blue), shown are 3rd, 50th, and 97th percentiles. **(A,C)** girls and **(B,D)** boys.

**Figure 7:**
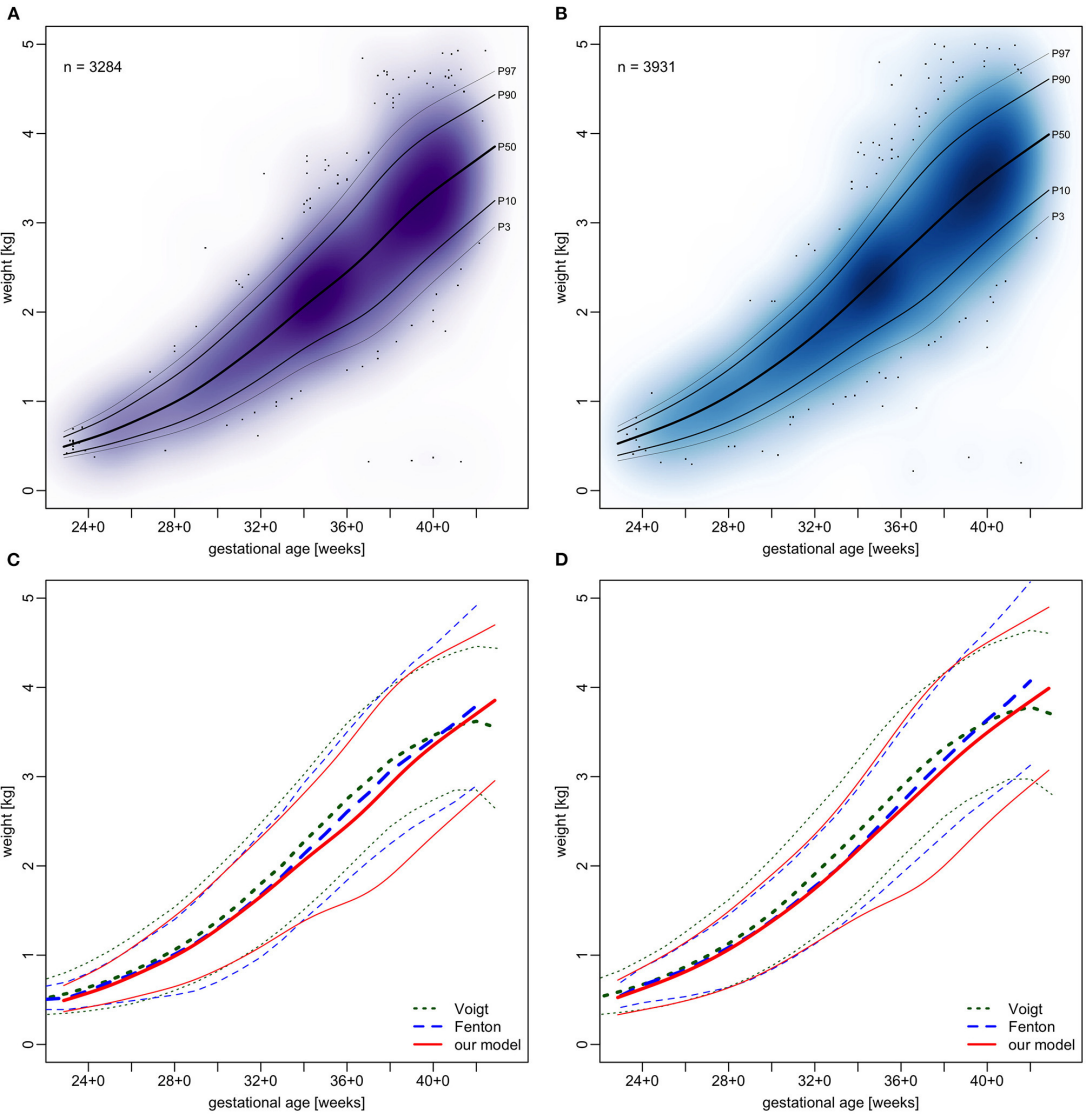
Charts of weight in relation to gestational age. **(A,B)** our sample with fitted models. **(C,D)** comparison of our models (red) with Voigt et al. (17) (green) and Fenton and Kim (13) (blue), shown are 3rd, 50th, and 97th percentiles. **(A,C)** girls and **(B,D)** boys.

We plotted OFC, CHL and BW from our sample in the same manner as EED and contrasted the resulting centiles with those by Fenton and Kim (13) and Voigt et al. (17) (see Figures 5–7). Compared to Voigt et al. (17) these relative differences were always positive, which means that Voigt et al. had larger values, with OFC values mostly within a 5% interval. This is most likely because they excluded multiple gestations while we kept them in our set. Interquartile ranges of relative differences to Fenton and Kim (13). OFCs and CHLs are crossing 0 and mostly fall within a 1% interval (see Supplementary Figure 4). Our centiles of OFC, weight and CHL closely resemble those by Fenton and Kim (13): The differences between 50th percentiles are minute and do not follow the same direction (i.e. one being consistently smaller than the other). Only the extreme percentiles show larger differences, especially for the term gestations. This may indicate that our infants have similar anthropometrics and allow using our new EED charts as an addition to the charts by Fenton and Kim (13).

While we relied on the exact gestational age as reported by the obstetrician, Fenton and Kim (13) rounded gestational ages down to completed weeks and compensated this by shifting the age forward by half a week. The latter has been suggested for low resource settings where one is less certain of gestational ages (18) which seems to work well since the resulting growth curves closely resemble each other with no relevant differences.

Fenton and Kim (13) combined various samples ranging from 623 to 14,146 infants with gestational ages below 30 weeks; our sample contains 786 considering the exact gestational ages reported by the obstetricians. The WHO growth charts for OFC were designed from data of 6,697 term neonates (19). While our sample is smaller since it comes from only one site, its size is well within the range of these studies. Our data are also far more recent at the time of submission than those used by Fenton and Kim (13). Longitudinal growth charts like the one by Ehrenkranz et al., who worked in settings very similar to ours, had some 1,700 patients (20). Earlier charts for small preterms were based on only 205 patients (21).

In spite of advances in optical (5, 6) and radiological (2, 3) three dimensional modeling cranial development is to date almost exclusively described by the planar OFC alone. This is likely due to the fact that it can be determined at minimal costs and with no safety concerns. In comparison with weight and CHL it has low levels of technical and observer bias (22). Due to the technical similarities we feel EEDs may be worth of these attributes as well. Such additional measurements may make help in estimating cranial volumes more precisely and allow for better assessments of multidirectional head growth. This could be particularly useful in the care of preterm infants. Absent OFC growth has been linked to neurodevelopmental impairment (23, 24), but the obviously deleterious disruption of important steps of brain development by preterm birth is not reflected in OFC growth (25).

Since only birth data were used, our charts were not biased by postnatal influences on head growth and shape. Deciding for a uniform approach, we did not consider the differences caused by perinatal factors such as vacuum extraction, breech birth, or caesarian section in our analyses.

The widespread use of CPAP prongs fixed to caps may constitute a factor which influences and possibly reduces OFC growth making a three-dimensional head growth assessment even more important. EED measurements may overcome this issue by providing an additional measurement of head growth in the third dimension, which is not as much influenced by caps. Which of the two EEDs presented here is more useful in following preterm infant head growth cannot yet be determined. Both versions are potentially useful, so correlation of longitudinal growth on the basis of the centiles presented here seem to be in order.

To better follow head growth we suggest using a head volume index (HVI) that combines OFC and vEED to one number that can be calculated easily. For this purpose, we have included percentile curves of HVI. The suitability of HVI as a surrogate of cranial volume to follow cranial growth during preterm infant care needs to be evaluated in future studies. Since we have not measured reference head volumes it is also not clear how HVI may be converted into an estimate of cranial volume.

## Conclusion

We herein suggest measuring transfontanellar and transvertical EEDs as additional parameters for following head growth and HVI to assess three-dimensional growth with a single number. We provide percentile graphs and LMS tables to improve understanding of head volume growth in preterm infants. The presented growth charts and LMS tables of transfontanellar and transvertical ear-to-ear distance as well as head volume index may become useful in clinical assessments. Further evaluations, including longitudinal analyses, are needed.

## Data Availability Statement

The datasets presented in this article are not readily available because release of the data has not been cleared by neither our institutional review board nor our data protection bureau. Requests to access the datasets should be directed to ascherl@medizin.uni-leipzig.de.

## Ethics Statement

The studies involving human participants were reviewed and approved by Institutional Review Board of the Medical Faculty at the University of Leipzig. Written informed consent to participate in this study was provided by the participants' legal guardian/next of kin.

## Author Contributions

UT and NA devised the study protocol and applied for the ethics commission vote. NA measured tEED and vEED of the healthy newborns. RA and NA gathered the charted data, performed all data analyses, and drafted the manuscript. All authors edited and approved the manuscript.

## Conflict of Interest

The authors declare that the research was conducted in the absence of any commercial or financial relationships that could be construed as a potential conflict of interest.

## Publisher's Note

All claims expressed in this article are solely those of the authors and do not necessarily represent those of their affiliated organizations, or those of the publisher, the editors and the reviewers. Any product that may be evaluated in this article, or claim that may be made by its manufacturer, is not guaranteed or endorsed by the publisher.

## Supplementary Material

Supplementary Table 1 LMS parameters of new models of occipitofrontal circumference (OFC) for girls.

Supplementary Table 2 LMS parameters of new models of occipitofrontal circumference (OFC) for boys.

Supplementary Table 3 LMS parameters of models of head volume index (HVI) for girls.

Supplementary Table 4 LMS parameters of models of head volume index (HVI) for boys.

Supplementary Figure 1 Plot of z-score distribution densities of excluded values versus included values. Filled red polygon shows the distribution of excluded values in its actual proportion to the included values.

Supplementary Figure 2 Gridded charts for ear-to-ear distances (EED) in relation to gestational age: transfontanellar EED (fEED), transvertical EED (vEED).

Supplementary Figure 3 Relative daily gains of the 50th percentile of occipitofrontal circumference (OFC), transfontanellar EED (fEED), and transvertical EED (vEED).

Supplementary Figure 4 Relative differences between the 50th percentiles of our new models and the corresponding percentiles by Voigt et al. and Fenton and Kim.

## Abbreviations

BW: birth weight
CHL: crown-heel length
EED: ear-to-ear distance
fEED: transfontanellar EED
GAMLSS: generalized additive models for location, scale, and shape
HVI: head volume index
LMS: Lambda Mu Sigma
NICU: neonatal intensive care unit
OBS: otobasion superius
OFC: occipitofrontal circumference
RKI: Robert Koch-Institut
vEED: transvertical EED
WHO: World Health Organization.
