# Supplementary material for: Charts and LMS Tables of Transfontanellar and Transvertical Ear-to-Ear Distances for Gestational Age
Source: Front Pediatr. 2022 May 4;10:838333. doi: 10.3389/fped.2022.838333 (PMC9116685; doi:10.3389/fped.2022.838333)
Supplement: Supplementary Table 1 — LMS parameters of new models of occipitofrontal circumference (OFC) for girls. [file Data_Sheet_1.docx]

Supplementary Table 1: LMS parameters of new models of occipitofrontal circumference (OFC) for girls.

| GA | P3 | P10 | P50=M | P90 | P97 | S | L | n |
| --- | --- | --- | --- | --- | --- | --- | --- | --- |
| 23 | 17.72 | 18.5 | 20.16 | 21.78 | 22.52 | 0.0633 | 1.2535 | 12 |
| 24 | 18.65 | 19.46 | 21.17 | 22.84 | 23.61 | 0.0622 | 1.2595 | 37 |
| 25 | 19.58 | 20.42 | 22.18 | 23.90 | 24.70 | 0.0612 | 1.2654 | 53 |
| 26 | 20.52 | 21.38 | 23.19 | 24.96 | 25.77 | 0.0602 | 1.2713 | 47 |
| 27 | 21.45 | 22.34 | 24.19 | 26.01 | 26.84 | 0.0592 | 1.2773 | 49 |
| 28 | 22.39 | 23.29 | 25.19 | 27.05 | 27.91 | 0.0582 | 1.2832 | 72 |
| 29 | 23.35 | 24.27 | 26.21 | 28.12 | 28.99 | 0.0572 | 1.2891 | 91 |
| 30 | 24.32 | 25.27 | 27.25 | 29.20 | 30.10 | 0.0563 | 1.2951 | 112 |
| 31 | 25.27 | 26.23 | 28.26 | 30.24 | 31.16 | 0.0553 | 1.3010 | 133 |
| 32 | 26.16 | 27.14 | 29.2 | 31.21 | 32.14 | 0.0544 | 1.3070 | 143 |
| 33 | 27.01 | 28.00 | 30.08 | 32.12 | 33.06 | 0.0535 | 1.3129 | 203 |
| 34 | 27.79 | 28.79 | 30.90 | 32.96 | 33.91 | 0.0526 | 1.3188 | 317 |
| 35 | 28.47 | 29.48 | 31.59 | 33.66 | 34.62 | 0.0517 | 1.3248 | 319 |
| 36 | 29.07 | 30.08 | 32.20 | 34.28 | 35.23 | 0.0508 | 1.3307 | 186 |
| 37 | 29.67 | 30.68 | 32.81 | 34.89 | 35.84 | 0.0500 | 1.3367 | 237 |
| 38 | 30.27 | 31.28 | 33.41 | 35.49 | 36.45 | 0.0492 | 1.3426 | 341 |
| 39 | 30.78 | 31.79 | 33.92 | 35.99 | 36.95 | 0.0483 | 1.3485 | 355 |
| 40 | 31.21 | 32.21 | 34.33 | 36.39 | 37.35 | 0.0475 | 1.3545 | 411 |
| 41 | 31.63 | 32.63 | 34.73 | 36.79 | 37.74 | 0.0467 | 1.3604 | 169 |
| 42 | 32.07 | 33.07 | 35.16 | 37.21 | 38.15 | 0.0459 | 1.3663 | 6 |

Supplementary Table 2: LMS parameters of new models of occipitofrontal circumference (OFC) for boys.

| GA | P3 | P10 | P50=M | P90 | P97 | S | L | n |
| --- | --- | --- | --- | --- | --- | --- | --- | --- |
| 23 | 18.21 | 19.02 | 20.8 | 22.66 | 23.55 | 0.0683 | 0.5554 | 10 |
| 24 | 19.18 | 19.98 | 21.73 | 23.54 | 24.41 | 0.0640 | 0.6217 | 32 |
| 25 | 20.14 | 20.93 | 22.67 | 24.45 | 25.30 | 0.0606 | 0.6880 | 56 |
| 26 | 21.06 | 21.87 | 23.62 | 25.41 | 26.26 | 0.0586 | 0.7542 | 60 |
| 27 | 21.93 | 22.77 | 24.59 | 26.42 | 27.29 | 0.0580 | 0.8205 | 83 |
| 28 | 22.72 | 23.62 | 25.56 | 27.52 | 28.43 | 0.0594 | 0.8868 | 83 |
| 29 | 23.45 | 24.44 | 26.56 | 28.69 | 29.69 | 0.0624 | 0.9530 | 96 |
| 30 | 24.23 | 25.3 | 27.58 | 29.86 | 30.93 | 0.0645 | 1.0193 | 132 |
| 31 | 25.20 | 26.29 | 28.61 | 30.92 | 31.99 | 0.0631 | 1.0856 | 156 |
| 32 | 26.36 | 27.40 | 29.61 | 31.80 | 32.81 | 0.0579 | 1.1518 | 208 |
| 33 | 27.53 | 28.50 | 30.55 | 32.57 | 33.50 | 0.0519 | 1.2181 | 213 |
| 34 | 28.53 | 29.46 | 31.42 | 33.34 | 34.23 | 0.0483 | 1.2844 | 356 |
| 35 | 29.22 | 30.18 | 32.20 | 34.18 | 35.09 | 0.0484 | 1.3506 | 330 |
| 36 | 29.67 | 30.71 | 32.89 | 35.01 | 35.99 | 0.0510 | 1.4169 | 225 |
| 37 | 30.05 | 31.17 | 33.51 | 35.77 | 36.80 | 0.0535 | 1.4832 | 326 |
| 38 | 30.55 | 31.69 | 34.06 | 36.34 | 37.38 | 0.0532 | 1.5494 | 369 |
| 39 | 31.17 | 32.26 | 34.52 | 36.70 | 37.69 | 0.0501 | 1.6157 | 450 |
| 40 | 31.86 | 32.86 | 34.94 | 36.93 | 37.84 | 0.0454 | 1.6820 | 477 |
| 41 | 32.59 | 33.5 | 35.38 | 37.19 | 38.02 | 0.0407 | 1.7483 | 261 |
| 42 | 33.28 | 34.13 | 35.87 | 37.55 | 38.31 | 0.0372 | 1.8145 | 7 |

Supplementary Table 3: LMS parameters of models of head volume index (HVI) for girls.

| GA | P3 | P10 | P50=M | P90 | P97 | S | L | n |
| --- | --- | --- | --- | --- | --- | --- | --- | --- |
| 23 | 3.22 | 3.52 | 4.29 | 5.26 | 5.80 | 0.1563 | -0.1401 | 1 |
| 24 | 3.99 | 4.37 | 5.33 | 6.51 | 7.16 | 0.1557 | -0.0846 | 2 |
| 25 | 4.74 | 5.19 | 6.33 | 7.73 | 8.49 | 0.1550 | -0.0291 | 9 |
| 26 | 5.44 | 5.97 | 7.28 | 8.87 | 9.72 | 0.1544 | 0.0265 | 8 |
| 27 | 6.12 | 6.72 | 8.20 | 9.98 | 10.92 | 0.1540 | 0.0820 | 4 |
| 28 | 6.83 | 7.51 | 9.17 | 11.15 | 12.19 | 0.1540 | 0.1376 | 11 |
| 29 | 7.61 | 8.39 | 10.27 | 12.48 | 13.63 | 0.1548 | 0.1931 | 23 |
| 30 | 8.48 | 9.37 | 11.51 | 13.99 | 15.28 | 0.1562 | 0.2486 | 22 |
| 31 | 9.42 | 10.43 | 12.86 | 15.65 | 17.09 | 0.1579 | 0.3000 | 18 |
| 32 | 10.41 | 11.56 | 14.29 | 17.40 | 18.99 | 0.1592 | 0.3597 | 26 |
| 33 | 11.45 | 12.74 | 15.78 | 19.21 | 20.94 | 0.1598 | 0.4152 | 31 |
| 34 | 12.51 | 13.94 | 17.28 | 21.01 | 22.88 | 0.1596 | 0.4708 | 56 |
| 35 | 13.54 | 15.11 | 18.72 | 22.71 | 24.69 | 0.1584 | 0.5263 | 64 |
| 36 | 14.56 | 16.23 | 20.05 | 24.21 | 26.26 | 0.1552 | 0.5818 | 35 |
| 37 | 15.61 | 17.35 | 21.28 | 25.50 | 27.56 | 0.1495 | 0.6374 | 58 |
| 38 | 16.71 | 18.47 | 22.42 | 26.59 | 28.62 | 0.1414 | 0.6929 | 94 |
| 39 | 17.84 | 19.59 | 23.47 | 27.51 | 29.46 | 0.1319 | 0.7484 | 111 |
| 40 | 18.99 | 20.71 | 24.47 | 28.36 | 30.21 | 0.1219 | 0.8040 | 113 |
| 41 | 20.19 | 21.86 | 25.49 | 29.20 | 30.95 | 0.1124 | 0.8595 | 52 |
| 42 | 21.40 | 23.02 | 26.52 | 30.06 | 31.73 | 0.1036 | 0.9150 | 2 |

Supplementary Table 4: LMS parameters of models of head volume index (HVI) for boys.

| GA | P3 | P10 | P50=M | P90 | P97 | S | L | n |
| --- | --- | --- | --- | --- | --- | --- | --- | --- |
| 23 | 2.44 | 3.01 | 4.30 | 5.70 | 6.38 | 0.2446 | 0.7529 | 0 |
| 24 | 3.17 | 3.83 | 5.37 | 7.08 | 7.94 | 0.2372 | 0.6532 | 2 |
| 25 | 3.93 | 4.67 | 6.44 | 8.46 | 9.49 | 0.2300 | 0.5534 | 6 |
| 26 | 4.72 | 5.53 | 7.51 | 9.83 | 11.03 | 0.2230 | 0.4536 | 10 |
| 27 | 5.56 | 6.44 | 8.62 | 11.23 | 12.6 | 0.2162 | 0.3533 | 14 |
| 28 | 6.46 | 7.41 | 9.79 | 12.69 | 14.25 | 0.2094 | 0.2521 | 15 |
| 29 | 7.47 | 8.48 | 11.05 | 14.26 | 16.01 | 0.2026 | 0.1507 | 20 |
| 30 | 8.58 | 9.66 | 12.43 | 15.94 | 17.89 | 0.1954 | 0.0521 | 23 |
| 31 | 9.78 | 10.93 | 13.89 | 17.69 | 19.82 | 0.1877 | -0.0364 | 23 |
| 32 | 11.04 | 12.26 | 15.39 | 19.43 | 21.7 | 0.1796 | -0.1021 | 33 |
| 33 | 12.32 | 13.61 | 16.89 | 21.10 | 23.46 | 0.1710 | -0.1315 | 37 |
| 34 | 13.61 | 14.96 | 18.36 | 22.67 | 25.06 | 0.1622 | -0.1221 | 41 |
| 35 | 14.89 | 16.30 | 19.80 | 24.14 | 26.51 | 0.1533 | -0.0815 | 34 |
| 36 | 16.16 | 17.62 | 21.20 | 25.52 | 27.84 | 0.1446 | -0.0166 | 35 |
| 37 | 17.40 | 18.90 | 22.53 | 26.79 | 29.04 | 0.1361 | 0.0665 | 67 |
| 38 | 18.59 | 20.12 | 23.76 | 27.93 | 30.08 | 0.1279 | 0.1619 | 89 |
| 39 | 19.70 | 21.25 | 24.86 | 28.91 | 30.96 | 0.1200 | 0.2650 | 146 |
| 40 | 20.75 | 22.30 | 25.86 | 29.76 | 31.70 | 0.1124 | 0.3718 | 128 |
| 41 | 21.78 | 23.33 | 26.82 | 30.56 | 32.40 | 0.1053 | 0.4796 | 71 |
| 42 | 22.81 | 24.34 | 27.75 | 31.35 | 33.09 | 0.0985 | 0.5876 | 2 |
